# *Hot topic:* Avian influenza subtype H5N1 in US dairy—A preliminary dairy foods perspective

**DOI:** 10.3168/jdsc.2024-0634

**Published:** 2024-09-30

**Authors:** Nicole H. Martin, Aljosa Trmcic, Samuel D. Alcaine

**Affiliations:** Department of Food Science, Cornell University, Ithaca, NY 14853

## Abstract

In February and March of 2024, an unusual illness began affecting dairy herds primarily in Texas and neighboring states. The causative agent of this illness was ultimately confirmed in late March 2024 to be a strain of highly pathogenic avian influenza H5N1 belonging to clade 2.3.4.4b. In the months following the emergence of this viral disease in cattle, infections have spread to at least 191 herds in 13 states at the time of this writing in August 2024, primarily through cattle and human movement. Surprisingly, early examination of raw milk samples from clinically affected animals indicated that the virus had an affinity for the mammary tissue, and viral shedding into raw milk occurred at high levels, exceeding 10^8^ log_10_ 50% tissue culture infectious dose (TCID_50_) in some cases. These high viral loads coupled with evidence that farm cats who consumed raw milk from clinically ill animals were infected and exhibited high mortality rates, raised concerns about the safety of the US milk supply for human consumption. To date, 4 cow-associated human infections have been reported, all from farm employees with direct contact with infected animals. Several parameters ultimately affect the theoretical public health risk from consumption of dairy products manufactured from a milk supply containing H5N1, namely (1) initial viral load, (2) persistence of H5N1 in raw milk, (3) viral inactivation through processing practices including pasteurization, and (4) human susceptibility and infectious dose. In the short period since the emergence of this disease in dairy cattle in the United States, research has begun to answer these critical questions, although our knowledge is still quite limited at this time. Here we review the literature available from the current H5N1 outbreak in US dairy cattle, as well as selected relevant literature from previous research in other animal agriculture sectors, that affect our current understanding of the parameters associated with the food safety risk of this disease in the US dairy supply chain.

## Highlights

• HPAI H5N1 viral loads in raw milk from affected animals are often very high.
• H5N1 virus is stable in raw milk at low temperatures for weeks.
• Pasteurization achieves a reduction in viral load, reducing risk to consumers.
• Infectious dose of H5N1 for humans through an oral consumption route is unknown.

## Graphical Abstract

**Figure:**
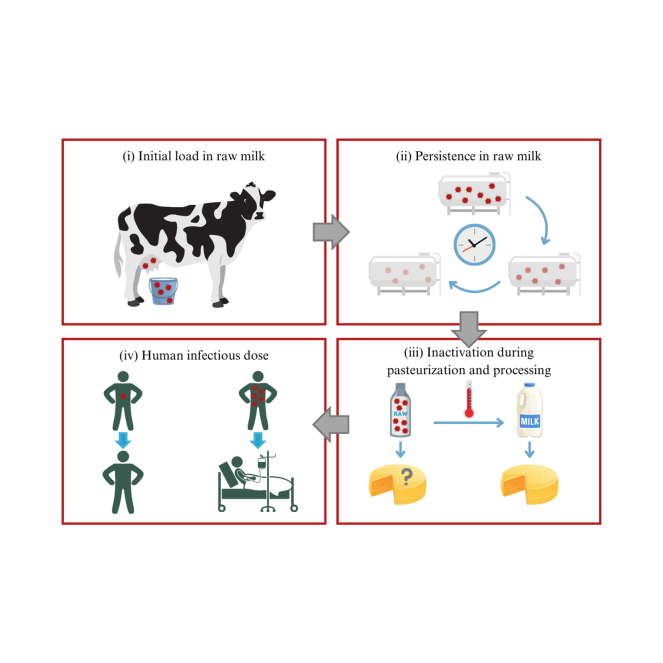
**Summary:** An emerging outbreak of highly pathogenic avian influenza (HPAI) in dairy cattle in the United States has raised questions about the safety of the milk supply for consumption. The theoretical transmission of avian influenza and other respiratory viruses through consumption of affected milk is dependent on several parameters, including what the initial level of infective virus is in raw milk, how long the virus maintains infectivity in milk over time, what impact pasteurization and other typical milk-processing parameters (e.g., acidification during cheesemaking) have on inactivation of the virus, and what dose humans would need to consume to become infected. Here, we discuss the research currently available from this outbreak, as well as select previous research, to understand these parameters and the associated food safety risk represented by this ongoing outbreak.

Avian influenza viruses are a subgroup of influenza A viruses, enveloped viruses within the *Orthomyxoviridae* family (Caserta et al., 2024). Avian influenza is classified as either highly pathogenic (**HPAI**) or low pathogenic (**LPAI**) by the severity of the disease caused in infected birds. The HPAI H5N1, so named for the hemagglutinin (H) and neuraminidase (N) surface glycoprotein variants present in this strain (Neumann et al., 2010), is further divided into clades and subclades. Avian influenza H5N1 clade 2.3.4.4b emerged in 2020 as the predominant subtype, ultimately spreading across the globe, and has caused devastating outbreaks in birds and sporadic spillovers into at least 30 mammalian species such as red foxes, harbor seals, and more (Green et al., 2023; Caserta et al., 2024). In the United States where H5N1 2.3.4.4b has been circulating since 2022, the impact of the ongoing outbreaks in poultry has been widespread, with over 90 million domestic birds culled in 48 states to date (CDC, 2024b), resulting in widespread egg and poultry shortages, and an economic impact estimated at $2.5 to $3 billion (Farahat et al., 2023).

In February and March 2024, dairy producers in Texas and neighboring states began to report unusual illnesses in lactating dairy cattle with primary symptoms including reduced DMI, reduced lactation, abnormal milk, mild respiratory symptoms, dehydration, and others (Caserta et al., 2024). By March 25, 2024, the causative agent of these illnesses was confirmed to be HPAI H5N1 clade 2.3.4.4b, and by the end of March, a total of 6 farms in 3 states were confirmed to have herds infected with this strain. In the several months (i.e., March 2024 through August 2024) since the emergence of this viral disease in dairy herds there have been a total of 151 herds in 12 states with confirmed cases (USDA, 2024), although it is likely that infections are more widespread than this official case count. This outbreak represents the first confirmed sustained transmission of HPAI H5N1 in a mammal species (Eisfeld et al., 2024).

While influenza viruses are respiratory viruses and there are no documented cases of humans contracting the virus through the consumption of contaminated food, there is a theoretical possibility that HPAI and other respiratory viruses can be transmitted through the consumption of contaminated food (O'Brien et al., 2021). The risk of foodborne transmission of HPAI H5N1 through milk and dairy products originating from affected herds in this outbreak is of particular concern as many of the affected dairy producers reported the deaths of outdoor domestic cats that had consumed raw milk from affected cows on the farm premises concurrently with the illnesses in cattle (Burrough et al., 2024). The risk of foodborne transmission of H5N1 from the current outbreak to humans depends on several parameters, including (1) initial infective viral load; (2) persistence of H5N1 in raw milk; (3) viral inactivation through processing practices including pasteurization, acidification, and so on; and (4) human susceptibility and infectious dose.

H5N1 is reported to be shed at high levels in milk from clinically symptomatic cows, ranging from 2.7 to 8.8 log_10_ tissue culture infectious dose (**TCID_50_**), for at least 3 d after clinical detection and at lower levels by infected, but asymptomatic, cows (Caserta et al., 2024). While the Pasteurized Milk Ordinance (**PMO**) requires that abnormal milk be discarded (USPHS/FDA, 2019), it is likely that presymptomatic, low symptomatic, and asymptomatic cows shedding virus could be milked and commingled with bulk raw milk from healthy cows. Indeed, PCR surveys of commercial pasteurized milk and bulk raw milk tanks have detected fragments of H5N1, suggesting that such events are common (Spackman et al., 2024a, Spackman et al., 2024b; Tarbuck et al., 2024 [unpublished data]). While PCR positives are indicative of the presence of the virus, raw milk studies have shown it is difficult to correlate quantitative real-time PCR threshold cycle values to the actual load of infectious virus (Caserta et al., 2024; Spackman et al., 2024a). Infectious virus has not been detected in commercially pasteurized milk samples (Spackman et al., 2024b; Tarbuck et al., 2024 [unpublished data]), but has been detected in commingled raw milk bulk tanks at levels ranging from 1.3 to 6.3 log_10_ 50% egg infective dose (**EID_50_**)/mL (Spackman et al., 2024a).

Early studies of bovine-associated H5N1 contaminated milk suggest that while viral loads decrease over time, infectious virus can persist for more than 5 wk when stored at 4°C (Guan et al., 2024). Although this is far beyond the time period that raw milk would remain viable for use in processing, this study highlights that the H5N1 strain from this outbreak remains stable for long periods of time at low temperatures. Other studies and models based on nonbovine H5N1 and influenza strains have also found that lower temperatures slow the rate of viral decay (Shahid et al., 2009; Mihai et al., 2011; Martin et al., 2018). The PMO states that raw milk must be cooled to 10°C within 4 h from the start of milking into an empty, clean bulk tank, and to 7°C, within 2 h after that, and that milk holding/storage tanks must be emptied at least every 72 h (USPHS/FDA, 2019). Based on these initial studies, it is likely that H5N1 would remain at appreciable levels in raw milk until pasteurization. Early studies of bovine-associated H5N1 on materials associated with dairy milking equipment found the that virus remained active for several hours, stressing the importance of cleaning and sanitization of equipment between raw milk loads at processing facilities to prevent cross-contamination and protect worker health (Le Sage et al., 2024). This is consistent with other environmental persistence studies of H5N1 in poultry settings (Wood et al., 2010). To date there are no studies evaluating the persistence of H5N1 in raw milk dairy products like cheese, though a study on influenza A virus found that it was not detectable by the end of the Cheddar cheesemaking process where infected milk was pasteurized at 63°C for 30 min before cheesemaking (Cliver, 1973). Some models of H5N1 persistence in water indicate an interaction between salinity and temperature (Martin et al., 2018), but it is unclear if salted cheeses aged at higher temperatures would have increased H5N1 inactivation rates. Future research must be conducted to fully determine the impact of pH, water activity, aging temperature, and other relevant parameters on the decay of H5N1 in dairy products.

The time and temperature for pasteurization of milk are required to be equivalent to 63°C for 30 min for vat pasteurization and 72°C for 15 s for continuous flow pasteurization, with an increase of 3°C in either method if the milk product has added sweeteners, has a fat content ≥10%, or a TS content ≥18% (e.g., ice cream mix, heavy cream, and so on; USPHS/FDA, 2019). H5N1 has previously been reported to be inactivated by pasteurization in eggs (Chmielewski et al., 2011, 2013); however, differences in pasteurization conditions and the food matrix might alter the thermostability of H5N1. Initial milk studies have found both cattle-associated H5N1 and other strains to be readily inactivated by conditions equivalent to vat pasteurization (Cui et al., 2024; Guan et al., 2024; Schafers et al., 2024 [unpublished data]), with an estimated 10 log_10_ TCID_50_/mL reduction after 2.5 min at 63°C (Kaiser et al., 2024). Some of these studies did find detectable levels of infectious H5N1 virus after treatments of at least 72°C for 15 s, but they were not performed using continuous flow systems, and therefore do not fully replicate commercial systems. An FDA study using such a system found no infectious virus after treatment at 72°C for 15 s and suggests that reductions of >12 log_10_ EID_50_/mL can be expected from industry-standard pasteurization practices (Spackman et al., 2024a). There are currently no published studies evaluating the impact of higher fat, solids, and sweeteners on H5N1 heat stability in dairy products. Furthermore, at the moment, there is a lack of data on sub-pasteurization time and temperature combinations (i.e., thermization, typically conducted at 57°C–68°C for at least 15 s) used by some raw milk cheesemakers to reduce risk of bacterial pathogens, and whether they are sufficient to reduce potential H5N1 loads.

In nondairy matrices, acidic and caustic conditions do promote H5N1 and other influenza virus decay at varying rates (Shahid et al., 2009; Wanaratana et al., 2010). Research is needed to determine what specific pH, hold times, and temperatures are required to reduce H5N1 loads to below detectable levels of infectious virus before disposal of contaminated milk. Likewise, there are several studies on the use of disinfectants, including chlorine, ozone, hydrogen peroxide, and formalin, and surfactants, including Tween and soaps, to inactivate H5N1 (Shahid et al., 2009; Lénès et al., 2010; Wanaratana et al., 2010; Pawar et al., 2015); however, the efficacy of these treatments in dairy matrices and the subsequent environmental impact needs to be evaluated. Other nonthermal methods, such as ultrafiltration and UV, show promise for H5N1 removal and inactivation in water (Lénès et al., 2010) and may have applications in some dairy byproduct and whey processing situations.

The final parameter to consider when assessing the risk of HPAI H5N1 transmission via dairy foods is the susceptibility of humans and infectious viral dose. The host range and virulence of the H5N1 virus at least in part depends on the activation pH of hemagglutinin, the protein on the virus surface involved in viral-cellular fusion and entry of the virus into the cell. Results of previous studies have shown that a change of hemagglutinin activation pH toward lower pH could be responsible for the adaptation and ability of this avian virus to infect mammal hosts. Specifically, a H5N1 virus mutant with hemagglutinin activation pH below 5.6 had greater growth and virulence in mice and ferrets compared with wild-type virus, whereas the opposite was true for growth and virulence in ducks and chickens (DuBois et al., 2011; Imai et al., 2012; Zaraket et al., 2013). In one of these studies, the lower hemagglutinin activation pH was also shown to be associated with higher acid stability of the virus (Zaraket et al., 2013), which could play an important role in the stability of the bovine strain of H5N1 in acidified dairy products such as cheese.

Since 1997 when the first confirmed human case of H5N1 infection occurred in Hong Kong (Claas et al., 1998), there have been 912 human cases reported globally of which 52% have been fatal. Primary risk factors for transmission from infected animals to humans include contact with sick or dead poultry, or preparation of sick birds for consumption (Van Kerkhove et al., 2011). From January 2022 through June 4, 2024, 29 sporadic human cases of H5N1 were reported with mortality reduced to 24% (CDC, 2024d; Garg et al., 2024). Globally reported human infections with H5N1 generally resulted in acute respiratory illnesses including fever, cough, runny nose, headache, and difficulty breathing (CDC, 2024d). Of the 14 infections reported in the United States since 2021 (4 infections in dairy workers and 10 infections in poultry workers), only one reported typical symptoms of acute respiratory illness associated with influenza virus infection, whereas the rest reported only mild symptoms characterized by fatigue and conjunctivitis (CDC, 2022, 2024a; Garg et al., 2024).

Human infectious dose for H5N1 virus is currently unknown and estimations of this dose must be made based on studies that either (1) evaluate similar viruses in humans or (2) evaluate the H5N1 virus specifically in animal models similar to humans. One research group summarized previous studies that report infectious doses for different influenza viruses based on trials using human volunteers (Yezli and Otter, 2011). The authors reported that large differences in reported doses and infection rates were due to various factors, including virus delivery, virus strain used, and susceptibility of the human volunteers; for example, reported doses for H1N1 virus that resulted in infection of all human volunteers in different studies ranged between 5 and 7 log_10_ TCID_50_ and similar to the proportion of infected volunteers challenged with 7 log_10_ TCID_50_ of H1N1 virus ranged between 58% and 100%. The lowest tested doses reported were for H2N2 virus, which caused infection in 44% and 50% of human volunteers when challenged with 0.7 and 1.6 log_10_ TCID_50_ of the virus, respectively. In a separate study, a research group used datasets from several studies where live attenuated reassortments of inﬂuenza A viruses (i.e., H1N1, H3N2) were used to evaluate infection using human volunteers and construct a dose-response curve for the wild-type of each virus (Watanabe et al., 2012). They determined that for wild-type H3N2 virus, the 10% infectious dose would be 1.3 log_10_ TCID_50_ (95% CI = 0.9–1.5 log_10_ TCID_50_), whereas they were not able to determine the final infectious dose for wild-type H1N1 virus based on analyzed datasets.

A recent study using a mouse model determined that an oral route of transmission of H5N1 in milk from infected cattle was possible when mice were inoculated with 50 µL (3 × 10^6^ plaque-forming units; PFU) of naturally contaminated bovine milk from the current outbreak (Guan et al., 2024). In another study, mice were orally inoculated with 25, 10, and 5 µL of naturally contaminated raw milk containing 1.3 × 10^4^ PFU/mL of H5N1, with 40%, 80%, and 100% survival, respectively (Eisfeld et al., 2024), again demonstrating in an animal model that there is a risk of oral transmission of this virus. While the level of human health risk associated with consuming raw milk from cows infected with H5N1 is currently still unknown (CDC, 2024c), it represents a high level of concern considering the high affinity of the virus for the mammary gland (Caserta et al., 2024; Guan et al., 2024), high loads of the virus found in raw milk from infected, but not necessarily symptomatic, cows (Caserta et al., 2024), and animal studies that have shown ingestion and inhalation of raw milk can be a potential route of infection in mammals (Guan et al., 2024).

Overall, the current state of research on the parameters affecting the theoretical foodborne transmission of H5N1 from milk to humans suggests that there is likely a low risk of foodborne transmission of H5N1 in pasteurized dairy products, even when milk from affected animals is inadvertently included in the milk supply, due to the majority of the milk supply being subjected to pasteurization in the United States. However, caution should be exercised concerning the consumption of unpasteurized (raw) milk and dairy products due to high viral loads and the persistence of H5N1 in milk for long periods of time. Due to the currently unknown infectious dose needed to cause human infection via oral ingestion, more research is needed to fully assess the risks associated with consumption of raw milk from affected cattle. This outbreak highlights the critical need for continued research and monitoring of emerging diseases that could have implications for the entire dairy system.
